# Untangling Tetanus: A Comprehensive Case Report Describing Its Diagnosis and Treatment

**DOI:** 10.7759/cureus.44702

**Published:** 2023-09-05

**Authors:** Özgür Söğüt, Hüseyin Ergenc, Yavuz Yigit

**Affiliations:** 1 Emergency Medicine, Department of Emergency Medicine, University of Health Sciences, Haseki Training and Research Hospital, Istanbul, TUR; 2 Emergency Medicine, Department of Emergency Medicine, Hamad Medical Corporation, Doha, QAT; 3 Blizard Institute, Queen Mary University, London, GBR

**Keywords:** immunity boosting measures, clinical manifestations, delayed diagnosis, vaccination, booster, tetanus

## Abstract

Tetanus, caused by a grave and potentially lethal bacteria, is a medical condition that severely affects the central nervous system and demands vigilant attention and comprehensive preventive measures to safeguard public health. The onset of this condition is sudden and characterized by the emergence of intense tonic muscle spasms, underscoring its critical nature. In Türkiye, around 50 cases are officially reported each year; however, it is widely suspected that numerous cases remain unreported, contributing to an even more significant impact. This report aims to shed light on a remarkable case involving a 24-year-old male patient. Despite having a complete vaccination history, this individual contracted tetanus and required intensive care and mechanical ventilation due to the severity of his tetanus infection. This case serves as a poignant reminder of the importance of booster administration, highlighting the significance of continued and timely reinforcement of vaccinations.

## Introduction

Tetanus, originating from Clostridium tetani's (C. tetani) tetanolysin toxin, is a severe central nervous system affliction. It manifests with a sudden onset and advances through tonic muscle spasms, ultimately proving fatal. C. tetani is a gram-positive bacterium that is motile, forms terminal spores, and lacks a capsule. These spores are plentiful in the intestinal systems of both humans and animals and are widely present in the soil [1,2]. Estimating the true global burden of tetanus is challenging due to the high prevalence of cases in developing countries with limited surveillance systems and early-stage vaccine programs [3]. Based on 2019 data released by the World Health Organization, a total of 14,745,000 tetanus cases were documented globally [4].

Inadequate immunization still remains the leading cause of tetanus [5]. In Türkiye, the tetanus vaccine is included in the childhood vaccination program, beginning in infancy, repeated in primary school, and followed by boosters at the age of 12-14 years in schools, with recommended 10-year intervals between shots [6]. Routine tetanus vaccinations are also provided to pregnant women and young men in military service. However, those who halt education after elementary school, do not receive military vaccination, drop out of service, or do not receive pregnancy care may miss the booster shots. Also, a decline in antitoxin levels over time can weaken immunity in vaccinated people [7].

In this report, we present the case of a young patient who, despite having been completely vaccinated, contracted tetanus and required intensive care with mechanical ventilation, emphasizing the significance of booster administration.

## Case presentation

A 24-year-old male patient presented to our emergency department with complaints of difficulty in opening the mouth and swallowing, painful muscle spasms in his back and neck, and sweating. The patient, who mentioned being pricked by a rusty nail in his left hand 10 days ago, had started experiencing pain and muscle contractions in his left hand just three days prior. Experiencing pain and spasms that had initially originated in the arm and later extended throughout the body, the patient had visited a healthcare facility due to discomfort in the hand. It was revealed that a tetanus vaccine had been administered at the center, but immunoglobulin had not been provided.

The patient mentioned receiving complete tetanus vaccinations before the contact with the nail. However, a detailed medical history indicated that his last tetanus vaccination had been at the age of seven years, and no subsequent interventions were reported. Upon examination, the patient was conscious, oriented, and cooperative. His pupils were miotic, his blood pressure measured 130/80 mmHg, and he had a respiratory rate of 28/min; the axillary temperature was 37.1 °C, and his pulse rate was 116/min. During the examination, observable risus sardonicus was noted, concomitant with nuchal rigidity and distressing generalized tonic contractions. With the lower extremities extended and upper extremities flexed, extensive rigidity and restricted voluntary movements were observed. The patient had no significant medical history, and he did not display alkalosis or acidosis in the arterial blood gas analysis. Additionally, the ionized calcium level was within the normal range. He did not report using any medication or substances, and the toxic panel conducted on the blood did not detect the presence of any substances.

The patient was administered 5 mg of intravenous diazepam, which led to partial improvement in his condition. No abnormalities were seen in CT and MRI. There were no pathological findings observed in the chest X-ray and electrocardiogram. The bedside ultrasound of the chest and abdomen, performed by the emergency medicine specialist, revealed no signs of pathology. There was a 0.5 cm x 0.5 cm erythematous area on the volar surface of the distal phalanx of the fourth finger of the patient's left hand. However, the wound area appeared clean, and there was no discharge.

The patient's laboratory findings revealed normal levels of hemoglobin (15 g/dL), hematocrit (43.9%), white blood cell count (8,460/mm^3^), platelet count (263,000/mm^3^), C-reactive protein (CRP) (3.4 mg/L), and calcium (10 mg/dL). However, creatine kinase (578 U/L, normal range: 0-171) and LDH (627 U/L, normal range: 0-247) values were elevated. Based on the patient's history and clinical findings, a diagnosis of tetanus was made.

In the emergency department, the patient received 3000 units of human tetanus immunoglobulin through intramuscular administration. Additionally, a 4 x 1500 mg metronidazole treatment was initiated. The patient was admitted to the ICU and continuously monitored in a subdued, dimly lit setting. The patient, whose symptoms persisted in the ICU, was intubated and sedated with 10 mg of midazolam, and 6 mg of vecuronium was administered as a muscle relaxant. To achieve sedation, an infusion of midazolam at a rate of 0.2 mg/kg/hour was commenced. A 4 mg/hour vecuronium infusion was initiated to alleviate muscle rigidity and prevent contractions. On the ninth day of his intensive care admission, the patient, whose contractions were evaluated by gradually decreasing sedation at planned intervals, had his endotracheal tube removed. After extubation, the patient was observed in the ICU for two more days. As his condition remained stable, he was subsequently transferred to the Infectious Diseases Service. Following an uneventful five-day observation period, the patient received his second tetanus vaccine and was discharged after arranging for a follow-up examination.

## Discussion

Every year, around one million cases of tetanus are reported worldwide, particularly in underdeveloped and developing countries, and approximately 200,000 of these cases result in fatalities [8,9]. The mortality rate among elderly patients and neonates can exceed 50%, while in developing countries, it reaches as high as 28 per 100,000 population [10]. At the time of his presentation, our patient indicated that his vaccination status was up to date. However, the detailed medical history revealed that his most recent vaccination had been at the age of seven years. We believe that the lack of subsequent booster administration to our patient had led to inadequate immunity and contributed to his contracting tetanus. Since the diagnosis and treatment of tetanus rely on clinical presentation and medical history, a detailed patient interview is essential. Moreover, there is a need to provide community education to address the gaps and misconceptions related to vaccinations.

In Türkiye, the tetanus vaccine has been a part of routine childhood immunizations since 1985 [11]. Booster doses are advised every 10 years, with the initial booster given to school-age children at the age of 12-14 years. Further boosters are provided to males during military service and to females during pregnancy [5].

Türkiye has achieved a success rate of 97% in primary vaccination coverage, placing it among the world's most successful countries in this regard [12]. However, in the 1990s, it was observed that the primary vaccination rate was around 90%, and, particularly in rural areas, vaccination rates were found to be low [13,14]. During the same period, the booster vaccination rates were around 32% [14]. Childhood primary vaccinations are mandatory in Türkiye. Booster vaccinations, on the other hand, are not mandatory, and issues can arise with regard to adult vaccinations. In this group, inadequate immunization can lead to fatal consequences, particularly after injuries. We believe that to address this issue, it is important to advocate for the implementation of a comprehensive government policy aimed at educating the public about the importance of booster immunization, particularly among the adult population.

In their study, Ergönül et al. identified insufficient tetanus antitoxin levels in 64-80% of the population aged 60 years and above and in 7% of the population aged 18-30 years [15]. In contrast, Kader et al. identified insufficient tetanus antitoxin levels in 37.1% of the population aged 18 years and older [11]. In the study by Tosun et al., inadequate immunity was found in 15% of the population aged 17-35 years and 44% aged 50-70 years [7]. Insufficient immunization among adults is a significant public health concern in our country and many other countries. In the study conducted by Pepersack et al. in Belgium, inadequate protection was identified in over 50% of elderly patients [16].

Tetanus is typically classified into four groups: generalized, localized, cephalic, and neonatal. The diagnosis is primarily based on clinical evaluation. Trismus, muscle rigidity, risus sardonicus, contractions triggered by visual and auditory stimuli, and any recent injury within the past three weeks strongly indicate generalized tetanus [17]. Localized tetanus mainly presents with muscle rigidity in the region around the wound. This rigidity can be mild or severe and may sometimes resolve spontaneously [18]. However, localized tetanus is often observed as a prodrome of generalized tetanus and can transform into generalized tetanus when a sufficient amount of toxin enters the central nervous system. Our case followed a similar course; the patient sought medical attention for localized tetanus symptoms and was administered tetanus toxoid at the healthcare center. However, as soon as tetanus diagnosis is suspected, preferably, human tetanus immunoglobulin should be administered intramuscularly at a dose of 3000-6000 IU [17].

Due to the wide range of clinical presentations and its rarity, diagnosing and treating tetanus can pose challenges. In addition, a significant portion of these patients present to emergency departments. The emergency department is a hectic place [19], and this can make obtaining detailed medical histories a challenging task. In the tetanus case report by Koruk et al., despite seeking medical help from various physicians, the patient was ultimately referred to the psychiatry clinic [20]. We believe that improving the knowledge level of healthcare personnel regarding tetanus will contribute to a decrease in tetanus cases.

Given the complexity of diagnosing and treating tetanus, which presents a wide range of clinical manifestations, the intricate interplay between the bacterium's neurotoxins and their effects on the nervous system underscores the multifaceted nature of the disease's progression and the challenges involved in its early and accurate diagnosis. It is crucial that healthcare professionals remain vigilant and informed about this condition. Documenting and communicating the results of a clinical trial varies based on the audience it is intended to target [21]. Therefore, just as medical interventions should be tailored to the specific needs of patients, the communication of scientific findings should be tailored to suit the comprehension levels and requirements of diverse readerships.

## Conclusions

While significant achievements have been made in childhood vaccinations in Türkiye, deficiencies related to booster administrations can still lead to individuals contracting tetanus even at a young age. Vaccination remains pivotal in the ongoing efforts against tetanus. All healthcare professionals should include this disease, diagnosed based on clinical evaluation and medical history, in their list of differential diagnoses. The successful management of this case was underpinned by the strategic implementation of early intubation, which notably averted potential respiratory complications and facilitated positive patient outcomes. We believe that healthcare personnel and the community should be made more aware of this rare and severe disease, which has a high mortality rate but can be almost entirely prevented with appropriate vaccination.
